# Biochemical Quality of Garlic Differs by Accession Provenance and Growing Season Rather than Morphotype

**DOI:** 10.3390/plants15192906

**Published:** 2026-09-23

**Authors:** Iva Bažon, Dean Ban, Martina Grdiša, Tvrtko Karlo Kovačević, Nikola Major, Anja Batel, Zlatko Šatović, Smiljana Goreta Ban

**Affiliations:** 1 Institute of Agriculture and Tourism, Karla Huguesa 8, 52440 Poreč, Croatia; iva@iptpo.hr (I.B.); dean@iptpo.hr (D.B.); tvrtko@iptpo.hr (T.K.K.); nikola@iptpo.hr (N.M.); abatel@iptpo.hr (A.B.); 2 Centre of Excellence for Biodiversity and Molecular Plant Breeding (CoE CroP-BioDiv), Svetošimunska Cesta 25, 10000 Zagreb, Croatia; mgrdisa@agr.hr (M.G.); zsatovic@agr.hr (Z.Š.); 3 Faculty of Agriculture, University of Zagreb, Svetošimunska Cesta 25, 10000 Zagreb, Croatia

**Keywords:** allicin, antioxidant capacity, biodiversity, bolting ability, morphotype, garlic accessions, total phenolic content

## Abstract

Preservation of plant genetic resources has become crucial for agricultural sustainability, particularly for vegetatively propagated crops like garlic. Modern agricultural practices, monoculture farming, environmental degradation, and climate change have led to genetic erosion, and there is growing interest in traditional garlic varieties, which may harbor superior traits. Despite being an asexually propagated crop, garlic exhibits a diverse range of ecotypes in phenotypic and agronomic characteristics. This study analyzed 77 garlic accessions from Istria and Kvarner, Dalmatia, Continental Croatia, and other countries, examining morphological traits alongside biochemical properties, namely allicin content, total phenolic content (TPC), and antioxidant capacity (with ferric reducing antioxidant power (FRAP) assay, and 2,2-diphenyl-1-picrylhydrazyl (DPPH) assay). Morphological analysis categorized the accessions into two distinct morphotypes, which did not differ significantly in any biochemical parameter. In contrast, accession provenance was associated with differences in TPC and FRAP, with the highest values in accessions from Istria and Kvarner; in 2018/2019, these reached 820.8 mg GAE/100 g DW (TPC) and 354.6 µmol TE/100 g DW (FRAP), significantly exceeding Continental Croatia (494.1 and 180.4, respectively). This regional pattern varied between the two vegetation years. DPPH and allicin content instead differed between the two vegetation years, with DPPH increasing markedly from 2018/2019 to 2019/2020 (from 358.1 to 892.2 µmol TE/100 g DW in QlG1 and from 380.6 to 836.3 µmol TE/100 g DW in QlG2). These results indicate that, in this collection, morphotype was not a reliable predictor of biochemical quality in garlic and underscore the importance of combining morphological and biochemical assessment in germplasm conservation and breeding programs.

## 1. Introduction

Garlic (*Allium sativum* L.) is one of the most cultivated and widely produced *Allium* species, ranked second after onion [1]. Its plantations are worldwide across different climate areas, ranging from temperate to subtropical areas [2]. According to the statistics of the Food and Agriculture Organization of the United Nations, the world production in 2019 amounted to 30,708,243 tonnes, with China being the leading country in garlic production with 28,220,105 tonnes [3]. Several review papers have demonstrated its important role in maintaining and promoting human health [4,5,6,7].

Garlic bulbs contain numerous bioactive compounds, with the most abundant being the organosulfur and phenolic compounds [8]. Phenolic compounds are one of the most commonly occurring plant secondary metabolites that contain one (phenol) or more (polyphenol) hydroxyl groups [9,10]. Phenolic compounds greatly contribute to the overall plant defense against herbivores, pathogens, and various abiotic stresses in the form of antioxidants [11]. Antioxidants are increasingly of interest to scientists and consumers due to their high capacity to scavenge free radicals [12]. According to the available literature, genotype, ecotype, environment, and year-to-year variations may significantly affect the phenolic content of garlic and consequently the overall antioxidant capacity [8].

Allicin is considered the dominant thiosulfinate in fresh garlic [13]. It is a sulphuric compound contributing significantly to garlic’s characteristic flavor and odor [14]. Mechanical damage to garlic tissue causes the enzyme alliinase to convert alliin, an odorless and biologically inactive allicin precursor, to allyl sulfenic acid (2-propenesulfenic acid). Two molecules of allyl sulfenic acid spontaneously condense to form allicin (diallyl thiosulfinate), the compound responsible for garlic’s characteristic pungency and much of its bioactivity [15]. Allicin is colorless and very unstable, thus rapidly decomposing into other volatile sulfur-containing compounds when bulbs are damaged [15]. In addition to imparting the well-known flavor and odor, allicin is praised for its antioxidant, antimicrobial, antibiotic, cardioprotective, and anticancer properties [16].

In recent decades, genetic erosion has increased due to modern agriculture, growth of monocultures, devastation of the environment, and climate change [17]. Preservation of plant genetic resources has become of key importance for agricultural production, especially for vegetatively propagated species such as garlic. Garlic germplasm collections have previously been screened for phytochemical traits, including allicin content, total phenolic content, and antioxidant capacity [18,19,20,21,22]. Morphological characterization based on standardized descriptors remains the first and most accessible step in evaluating plant genetic resources, providing the phenotypic framework against which biochemical and molecular variation can be interpreted [23]. Internationally recognized *Allium* descriptors [24] have been applied to document phenotypic diversity across germplasm collections worldwide, revealing substantial variation in bulb, clove, and foliar traits [25,26]. Such characterization has proven informative both for distinguishing genotypes and for relating morphology to geographic origin and end-use quality. Some studies have combined morphological descriptors with multivariate analysis to structure diversity and to link phenotype with cultivation environment [25,27], while others have paired morphological traits with molecular markers to resolve genetic divergence among cultivars [28]. Assessing garlic germplasm at both the morphological and the biochemical level offers a more complete basis for conservation and breeding than either approach alone, particularly for a vegetatively propagated crop whose diversity is associated with clonal maintenance and local adaptation.

Therefore, the objective of this study was two-fold: first, to characterize the morphological diversity of a broad garlic collection using standardized descriptors; and second, to test whether morphotype, accession provenance, and growing season are associated with the biochemical profile of the accessions, namely allicin content, total phenolic content, and antioxidant capacity. Establishing whether readily scored morphological traits, in particular the ability to bolt, can serve as a proxy for biochemical quality has direct implications for how germplasm collections are screened, managed, and exploited in breeding.

## 2. Results

### 2.1. Morphological Traits

Investigated garlic accessions exhibited 1 of the 4 shapes of mature dry bulbs (Ql1): flat globe, rhomboid, broad oval, and globe (Figure 1).

In the case of the mature garlic bulb shape (Ql2), samples exhibited one of the following shapes: circular with a prominent basal plate, heart-shaped with a retracted basal plate, or broadly oval with an even basal plate. According to the descriptors for the outer skin color of compound bulbs (Ql3), the bulb color was described as either white, cream, light violet, or with white stripes. Regarding the skin color of cloves (Ql4), samples exhibited a range of colors: white, yellow with brown, brown, red, or violet. Investigated samples exhibited 1 out of 5 following bulb structure types (Q15): regular multi-fan groups, regular two-fan groups, regular multi-cloved radial, regular quadruple, and irregular (Figure 2).

The shape of the compound bulb (Ql6) in horizontal section was either circular or elliptical. The investigated garlic accessions were described as either having the ability to flower (bolt) or not (Q17).

Shannon diversity parameters for the seven qualitative traits are presented in Table 1. Total diversity (H_T_) varied considerably among traits, ranging from 0.963 (95% CI: 0.87–1.00) for ability to flower (Q17) to 1.924 (95% CI: 1.73–2.05) for clove skin color (Q14). High total diversity was also observed for outer bulb skin color (Q13; H_T_ = 1.889) and bulb structure type (Q15; H_T_ = 1.867), whereas the shape of the compound bulb in horizontal section (Q16) and ability to flower (Q17) exhibited the lowest levels of diversity. The proportion of diversity attributable to differences among accessions varied substantially among traits. The lowest among-accession diversity was observed for Q16 (36.62%; 95% CI: 28.00–45.10%), followed by shape of mature dry bulbs (Q11; 44.38%; 95% CI: 38.79–49.66%) and shape of mature garlic bulb (Q12; 47.12%; 95% CI: 39.56–53.86%). In contrast, the highest among-accession diversity was recorded for Q17 (86.23%; 95% CI: 80.02–91.42%), indicating that most variation in this trait occurred between rather than within accessions. High levels of among-accession diversity were also observed for Q15 (72.35%) and Q13 (62.45%). For all traits, the majority of diversity was distributed within accession provenance groups rather than among accession provenance groups. The proportion of diversity attributable to differences among accession provenance groups ranged from only 1.68% for Q11 to 11.44% for Q17. These findings indicate weak regional differentiation across all analyzed traits and suggest that most phenotypic variation is maintained within, rather than among, accession provenance groups. Bootstrap-derived 95% confidence intervals confirmed the overall robustness of the diversity estimates. Confidence intervals for the proportion of diversity among accession provenance groups were relatively broad, particularly for Q17, reflecting the low magnitude of regional differentiation. Nevertheless, for all traits, the regional component remained substantially smaller than the corresponding within-accession provenance groups component.

Regional diversity partitioning results are presented in Table S2. The relative contributions of among- and within-accession diversity varied among traits and accession provenance groups. Bulb structure type (Q15) and ability to flower (Q17) exhibited the highest proportions of diversity among accessions, whereas the shape of the compound bulb in horizontal section (Q16) was characterized by a predominance of within-accession diversity. Bootstrap-derived confidence intervals indicated moderate uncertainty for most estimates but did not alter the overall patterns of diversity partitioning observed across traits.

The frequencies of qualitative trait states observed among the 10 plants of each accession were subjected to principal component analysis (PCA) (Figure 3). The first two principal components (PC1 and PC2) jointly explained 38.33% of the total variability. K-means cluster analysis was subsequently performed based on the same accession-level trait-state frequencies. The ABC procedure selected two clusters (K = 2), with a Gap statistic of 0.4029. Accordingly, the 77 garlic accessions were separated into two groups, QlG1 and QlG2, representing distinct morphotypes.

Group QlG1 comprised 46 accessions and was characterized by yellow and light-brown clove skin color (Ql4_2), an irregular bulb structure type (Ql5_6), an elliptic shape of the compound bulb in horizontal section (Ql6_2), and the inability to flower (Ql7_0). Group QlG2 comprised 31 accessions and was characterized by violet clove skin color (Ql4_5), a regular two-fan group bulb structure type (Ql5_2), a circular shape of the compound bulb in horizontal section (Ql6_1), and the ability to flower (Ql7_1) (Figure 3).

### 2.2. Biochemical Properties

Total phenolic content (TPC), ferric reducing antioxidant power (FRAP), 2,2-diphenyl-1-picrylhydrazyl (DPPH) assay, and allicin content were analyzed with respect to morphological group, accession provenance, and vegetation year, together with all interactions (Table 2). The mixed-model analysis revealed different patterns of variation among the biochemical parameters. For TPC, a significant effect of accession provenance was detected (*p* < 0.001), together with a significant Accession provenance × Year interaction (*p* < 0.001). Because of the significant Accession provenance × Year interaction, the effect of accession provenance was examined separately for each vegetation year. In 2018/2019, TPC differed significantly among accession provenance groups (*p* < 0.001), with the highest value observed in Istria (820.8 ± 31.6 mg GAE/100 g DW), whereas the values in Continental Croatia, Dalmatia and non-Croatian accessions were lower (494.1 ± 47.9, 593.8 ± 45.6 and 541.8 ± 31.0 mg GAE/100 g DW, respectively) (Figure 4). In 2019/2020, the effect of accession provenance groups was not significant, with TPC values ranging from 511.1 ± 51.9 mg GAE/100 g DW in Continental Croatia to 629.9 ± 33.6 mg GAE/100 g DW in non-Croatian accessions.

FRAP was significantly affected by accession provenance (*p* < 0.01) and showed a significant Accession provenance × Year interaction (*p* < 0.01) (Table 2). Simple-effects analysis indicated significant differences among accession provenance groups in both vegetation years. In 2018/2019, FRAP was highest in Istria (354.6 ± 14.9 µmol TE/100 g DW), which differed significantly from Continental Croatia (180.4 ± 22.6 µmol TE/100 g DW), while Dalmatia and non-Croatian accessions showed intermediate values (Figure 4). In 2019/2020, the highest FRAP value was observed in non-Croatian accessions (319.1 ± 17.9 µmol TE/100 g DW), whereas the values in Istria, Dalmatia and Continental Croatia were lower (243.0 ± 18.2, 250.4 ± 26.3 and 226.2 ± 27.6 µmol TE/100 g DW, respectively). Thus, the accession provenance pattern of FRAP differed between the two vegetation years.

DPPH was significantly affected by vegetation year (*p* < 0.001) and showed a significant Group × Year interaction (*p* < 0.05). Therefore, the effect of year was examined separately within each morphological group. DPPH increased markedly from 2018/2019 to 2019/2020 in both morphological groups, from 358.1 ± 20.8 to 892.2 ± 20.8 µmol TE/100 g DW in QlG1 and from 380.6 ± 13.7 to 836.3 ± 13.7 µmol TE/100 g DW in QlG2 (both *p* < 0.001) (Figure 4). Although the magnitude of the increase differed between the two groups, the groups did not differ significantly within either vegetation year.

Allicin concentration was significantly affected by vegetation year (*p* < 0.001), whereas no significant effects of morphological group, accession provenance, or their interactions were detected (Table 2). Allicin concentration increased from 136.2 ± 15.2 mg/100 g DW in 2018/2019 to 207.5 ± 15.2 mg/100 g DW in 2019/2020, with the two years differing significantly according to Tukey’s HSD test.

Spearman’s rank correlations among TPC, FRAP, DPPH, and allicin content were calculated separately for each vegetation year (Table 3), using the same 77 accessions in each year. In both years, TPC and FRAP showed strong and positive correlations, with the strongest association observed in 2018/2019 (ρ = 0.892, 95% CI: 0.88–0.96, *p* < 0.001) compared with 2019/2020 (ρ = 0.669, 95% CI: 0.57–0.79, *p* < 0.001). TPC and FRAP were also positively correlated with DPPH radical-scavenging capacity in both years, although these associations were weaker than the TPC–FRAP relationship. In 2018/2019, the correlations were ρ = 0.456 (95% CI: 0.27–0.63, *p* < 0.001) for TPC–DPPH and ρ = 0.492 (95% CI: 0.29–0.61, *p* < 0.001) for FRAP–DPPH. In 2019/2020, the corresponding correlations were ρ = 0.360 (95% CI: 0.14–0.52, *p* = 0.003) and ρ = 0.550 (95% CI: 0.32–0.71, *p* < 0.001), respectively.

Associations involving allicin differed between the two vegetation years. In 2018/2019, allicin was not significantly correlated with TPC (ρ = −0.085, 95% CI: −0.33–0.14, *p* = 0.929) or FRAP (ρ = 0.040, 95% CI: −0.22–0.22, *p* = 0.727). Its correlation with DPPH was also not significant after Holm correction (ρ = −0.233, 95% CI: −0.35–0.05, *p* = 0.123). In 2019/2020, allicin showed weak but significant positive correlations with TPC (ρ = 0.373, 95% CI: 0.12–0.49, *p* = 0.002) and FRAP (ρ = 0.382, 95% CI: 0.25–0.61, *p* = 0.002), whereas its correlation with DPPH was weak and non-significant (ρ = 0.182, 95% CI: −0.04–0.38, *p* = 0.114).

## 3. Discussion

Within this study, 77 accessions were described using ECPGR descriptors for *Alliums* (*Allium* spp.) based on vegetative and inflorescence descriptors (Table S1). Shannon’s diversity index was used as an indicator of variation in garlic traits, either within accessions or among accessions [22,25]. According to Khosa et al. (2014), a high level of diversity for Indian accessions was observed in traits such as bulb skin color and shape of mature dry garlic bulbs, while a low level of diversity was observed in the ability to flower [29]. In our study, both traits, bulb skin color (Ql3) and shape of mature dry garlic bulbs (Ql1), were also found to exhibit a high level of diversity according to the value of total diversity (Table 1). Skin color of the clove (Ql4), bulb structure type (Ql5), shape of the compound bulb in horizontal section (Ql6), and ability to flower (Ql7) separated our accessions into two groups, with ability to flower having the strongest impact on differentiation of accessions among investigated qualitative traits.

Wang et al. (2014) clustered Chinese accessions into 2 major groups according to the flower ability: bolting and non-bolting garlics [22]. Division of garlic based on the ability to flower was beneficial to breeders interested in the production of flower scapes for fresh consumption, for true seed production, and fertility restoration programs [22]. However, further study is needed concerning garlic inflorescence since some accessions may be described as semi-bolters according to the ability to produce a scape, while in many bolting accessions, flowers can be found to be sterile [30].

According to studies by Kamenetsky et al. (2004, 2005), the formation of flowers in garlic is mostly under genetic control, and it occurs under short photoperiod and low temperature [31,32]. Since the accessions investigated in our study were planted in autumn, it suggests that the conditions for flower differentiation were met, allowing us to observe the differences in bolting ability among the accessions.

Research indicates that geographical conditions have accelerated the selection of garlic. Studies suggest that garlic from regions with harsh, cold winters and heavy snowfall has evolved to be non-bolting due to these severe agroclimatic conditions [30]. Also, garlic cloves may have ceased bolting because farmers worked to avoid reduced bulb yields [26]. A hypothesis was proposed that garlic has evolved from fertility to sterility and from a complete bolting type to a non-bolting type through an incomplete bolting type [30]. Both non- and incomplete-bolting traits are presumably the products of adaptation to unfavorable climatic conditions [19,26]. For all seven qualitative traits, the majority of morphological diversity (≈88–98%) was partitioned within rather than among accession provenance groups. The share attributable to differences among accessions varied by trait, and it was the highest for bulb structure type and flowering ability, and the lowest for bulb shape, indicating that accession provenance is a poor predictor of morphological identity while individual accessions remain the meaningful unit of diversity.

Clove coloration is considered one of the most important traits in garlic variety differentiation. One such example is purple garlic, which exhibits one of the highest total phenolic contents [19]. Depending on the purpose of use and market preference, the accessions in our collection can be distinguished according to their clove color into yellow and light brown accessions (Ql4_2) and violet accessions (Ql4_5). Jabbes et al. (2012) reported a similar division based on clove color, differentiating light-colored (white, pink, brown) garlic accessions from colored (violet and purple) ones [20].

Poljuha et al. (2021) have reported that the accessions from Istria and Kvarner (R2) had the highest share of private genotypes, as well as the highest private genotypic richness, indicating the potential of this area as a valuable source of genetic material for breeding [33]. This is further corroborated by the results from our study, in which accessions from Istria and Kvarner (R2) had a higher value of diversity among accessions within the accession provenance groups (H_R_) in several traits such as outer skin color (Ql3), clove color (Ql4), shape of the compound bulb in horizontal section (Ql6), and ability to flower (Ql7) compared to the remaining Croatian regions (Table S2). Notably, the Istrian accessions that exhibited the highest among-accession morphological diversity also showed the highest mean TPC (Figure 4), indicating that this region is a valuable source of both morphological and phytochemical variation for conservation and breeding aimed at antioxidant quality.

The first group of garlic accessions (QlG1) in our study may be equivalent to variety *sativum*, namely to the horticultural types “Artichoke” and “Silverskin”, according to the descriptions provided by Engeland (1991) [34]. Belonging to the *sativum* variety, the mentioned horticultural types are considered non-bolters, which is in line with our results, as the cultivars in QlG1 were characterized by not having the ability to bolt. In addition, the clove skin color of accessions in QlG1 was either yellowish or light brown, which is in accordance with the description of both “Artichoke” and “Silverskin” types, whose clove skin color is typically yellow-white and off-white with copper veins, respectively [34]. The second group of garlic accessions (QlG2) in our study was characterized by bolting ability, violet clove skin color, regular two-fan-group bulb structure, and circular shape of the bulb, all of which are also characteristic of both “Rocambole” and “Continental” horticultural types. The classification of accessions in our study is in accordance with the descriptions reported by Engeland [34].

When comparing garlics from the Argentinian collection based on flowering behavior, bolting garlics had significantly higher TPC than non-bolting garlics [19]. In contrast, Hirata et al. (2016) did not observe any significant difference in TPC between 4 types of garlic: bolters that produced mainly flowers, bolters producing mainly bulbils, incomplete bolters, and non-bolting garlic, although 3 accessions originating from the New World described as bolters producing mainly bulbils had exhibited a significantly lower TPC [26]. In our study, no significant difference in TPC between the bolting and non-bolting accessions was observed. This is in accordance with the study by Hirata et al. (2016) [26], but opposite to what Barboza et al. (2020) reported [19].

According to the literature, the presence of scape suggests competition between bulb and inflorescence development in bolting plants [35]. Usually, top sets are produced in the inflorescence rather than flowers, which might be due to millennia of garlic selection and certain hormonal changes [36].

Instead of morphotype, the biochemical variation in our study was structured mainly by accession provenance and growing season, as shown by the significant interaction effects. TPC and FRAP were affected by the accession provenance × year interaction (both *p <* 0.001), indicating that the accession provenance differences in phenolic content and reducing capacity were not consistent across the two vegetation years. DPPH showed a significant group × year interaction (*p <* 0.05), meaning the effect of vegetation year on radical scavenging capacity differed between the two morphological groups (Table 2). The interaction between genotype and accession provenance was shown to be a major contributor to the variability of TPC and antioxidant capacity, according to Beato et al. (2011) [37]. The same authors suggested that garlic cultivars responded differently to various environmental stimuli, resulting in significant changes in agronomic traits and biochemical profiles [37]. Overall, the phenolic-related measures (TPC, FRAP, and to a lesser extent DPPH) were consistently and positively interrelated across both years, whereas the relationship between allicin content and the antioxidant parameters was weak and inconsistent, changing in both magnitude and sign between the two vegetation years.

A study by Wongsa et al. (2016) compared the bolting (hardneck) and non-bolting (softneck) garlics in terms of allicin content [38]. It was observed that the softneck varieties did not statistically differ in allicin content from the hardneck varieties [38]. This is in accordance with the results of our study, since no significant difference in allicin content between the morphological groups was observed (Table 2). In addition, results of both our study and the aforementioned studies are further corroborated by the reports by Wang et al. (2014) [22] and Baghalian et al. (2005) [39], which concluded that there is no significant correlation between morphological traits and allicin content.

Almost all accessions originating from Continental Croatia, Istria and Kvarner, and Dalmatia are landraces for which selection was not carried out. Few of them were listed on the List of Varieties of the Republic of Croatia (Brgudski syn Benkovački, Cerićki, Istarski crveni, Podravski, Slavonski and Šokac) as conservation varieties. Therefore, lack of selection for specific traits among Croatian landraces may have caused the lack of difference in allicin content between the 2 morphological groups. Taken together, the clear morphological separation of the two groups combined with their lack of biochemical differentiation shows that morphological and biochemical characterization provide complementary rather than interchangeable information, and that selection for a desired biochemical profile cannot rely on morphotype alone.

In both vegetation years, a significant positive correlation was found between TPC and FRAP and DPPH assays, and a significant correlation was observed between the antioxidant assays as well (Table 3). The correlation analysis performed on 43 cultivars from Chinese germplasm in a study by Chen et al. (2013) showed a significant positive correlation between TPC and both FRAP and DPPH assays, as well as between FRAP and DPPH assays [40]. Avgeri et al. (2020) observed a significant positive correlation between TPC and FRAP antioxidant capacity of Greek landraces and between the imported garlic genotypes as well [18]. Santas et al. (2008) reported the same significant positive correlation found in onions [41]. All the aforementioned studies are further corroborated by the results of our study, thus indicating that phenolics are mainly responsible for the antioxidant and antiradical activity in garlic [42].

Content of garlic organosulfur compounds, whether measured as total thiosulfinate content or measured individually as alliin (a precursor of allicin), was found to positively correlate with the DPPH assay [43]. In some cases, a significant positive correlation was observed between the organosulfur content and FRAP antioxidant capacity of garlic [44]. In the case of the vegetation year 2018/2019, our results of correlation analysis between allicin content and both FRAP and DPPH antioxidant capacities were opposite to the results of the studies by Kim et al. (2018) and Soto et al. (2016), as the correlation between allicin and FRAP was found to be not significant, and the correlation between allicin and DPPH was significant but negative (Table 3) [43,44]. On the other hand, the results of correlation analysis for the vegetation year 2019/2020 showed a significant positive correlation between FRAP and allicin, while no significant correlation between allicin and DPPH was observed. The correlation between allicin content and the antioxidant assays differed between the two vegetation years (Table 3). In this study, the two vegetation years assessed differed in autumn planting date by a few weeks; that means the year-to-year variation might reflect both seasonal conditions and planting date. Multi-site, multi-year trials would help to more extensively separate genotypic, provenance, and environmental effects.

## 4. Materials and Methods

### 4.1. Experimental Setup and Plant Material

A total of 77 garlic accessions from different accession provenance groups were included in the study. Fifty-five accessions were collected from Croatia: 13 accessions from Continental Croatia (R1), 22 from Istria and Kvarner (R2), and 20 from Dalmatia (R3), and 22 accessions were collected from foreign countries (R4): Australia, Czech Republic, France, Finland, Greece, Hungary, Italy, Kazakhstan, Netherlands, Norway, Romania, Russia, Slovenia, and Spain (Table S1). In the period from 2014 to 2018, plant material was collected from local producers (traditional small farms) and institutions maintaining garlic collections. Following collection, all accessions were maintained and propagated in the experimental field at IPTPO prior to the start of the experiment. Consequently, the bulbs used in the two experimental years (2018/2019 and 2019/2020) were produced under common conditions at Poreč, reducing residual effects of the original collection environments. In each experimental year, representative bulbs of each accession were selected from the garlic collection maintained at IPTPO and used as planting material. The non-Croatian group (R4) pools 22 accessions from 14 countries spanning diverse geographic and climatic conditions, unevenly sampled across them. Although all accessions were grown under common conditions at IPTPO, R4 remains genetically and geographically heterogeneous by virtue of its diverse origins. It should therefore be treated as a pooled comparison group rather than a coherent provenance region.

Bulbs were planted in the experimental field of the Institute of Agriculture and Tourism (Poreč, Croatia, N 45°13′20.30″, E 13°36′6.49″). The samples were grown on *terra rossa* soil. Common agricultural practices for garlic growing were applied according to Lešić et al. [45]. Herbicides were not applied as the weeds were removed manually. Complex fertilizers were applied before planting, i.e., NPK 7:20:30 (Petrokemija Kutina, Kutina, Croatia) at 300 kg/ha. Every year, per hectare, 42 kg of N, 120 kg of P_2_O_5_, and 180 kg of K_2_O were added to the soil. In addition, during vegetation, garlic was sprayed against garlic fly, *Suillia lurida*, with the insecticide Decis 2.8 EC (deltamethrin, Bayer CropScience AG, Monheim am Rhein, Germany) at a rate of 0.1 L/ha. Mineral N fertilizer, KAN (Petrokemija Kutina), was added to the soil at 250 kg/ha. A minimum of 40 uniform cloves per accession were planted using a completely random design, at a plant density of 38 plants/m^2^ in 4-row beds, at a planting distance of 20 cm between rows and 13 cm in a row. Planting depth was 5 cm. In each vegetation year, all accessions were planted on the same day: 15 October 2018 for the 2018/2019 season and 26 November 2019 for the 2019/2020 season. Bulbs were harvested at the end of June or the beginning of July, when the lower leaves had died off. No accession was harvested later than early July. After harvest, bulbs were cured in shade for three weeks at room temperature, cleaned, and stored under the same conditions until replanting in autumn. As garlic is propagated clonally, the same clonal accessions were evaluated in both vegetation years, with each year’s planting material produced from bulbs of the same accession grown at IPTPO.

Each accession was grown as a single field plot per vegetation year, arranged in a completely randomized design. The experiment was rainfed; no supplemental irrigation was applied. Representative bulbs with no visible symptoms of plant diseases or physiological damage were sampled for biochemical analyses. A two-year field experiment (vegetation years 2018/2019 and 2019/2020) was carried out. Growing-season weather conditions were characterized using monthly precipitation, air temperature, and relative humidity recorded at the IPTPO meteorological station over both experimental seasons (Figure S2). The two seasons differed mainly in the amount and distribution of rainfall: the 2018/2019 season had a rainy late spring, whereas the 2019/2020 season was much drier in the spring. Air temperature followed a similar seasonal course in both years, with only minor year-to-year differences, so the seasons were more alike in temperature than in precipitation. Relative humidity was likewise broadly comparable between years, tracking the seasonal pattern and reaching its highest values in the wetter autumn and winter months. Detailed monthly values are provided in Figure S2.

### 4.2. Morphological Analysis

Ten garlic bulbs from each accession were described according to ECP/GR descriptors for *Allium* (*Allium* spp.) based on vegetative and inflorescence descriptors which were developed within the International Plant Genetic Resources Institute [24]. Traits, along with the IPGRI Descriptor Numbers and their corresponding abbreviations, are presented as follows:

- Shape of mature dry bulbs (7.1.11) (Flat globe Ql1_2, Rhomboid Ql1_3, Broad oval Ql1_4, Globe Ql1_5);
- Shape of mature garlic bulb (7.1.12) (Circular, basal plate prominent Ql2_1, Heart-shaped, basal plate retracted Ql2_2, Broadly ovate, basal plate even Ql2_3);
- Outer skin color of compound bulb (7.1.16.1) (White Ql3_1, Cream Ql3_2, White stripes Ql3_4, Light violet Ql3_5, Violet Ql3_6, Dark violet Ql3_7);
- Skin color of the clove (7.1.16.2) (White Ql4_1, Yellow and light brown Ql4_2, Brown Ql4_3, Red Ql4_4, Violet Ql4_5);
- Bulb structure type (7.1.20) (Regular multi-fan groups (or multi-shelled) Ql5_1, Regular two-fan groups Ql5_2, Regular multi-cloved radial Ql5_3, Regular quadruple Ql5_4, Irregular Ql5_6);
- Shape of the compound bulb in horizontal section (7.1.21) (Circular Ql6_1, Elliptic Ql6_2);
- Ability to flower (7.2.1) (No Ql7_0, Yes Ql7_1).

### 4.3. Biochemical Analysis

#### 4.3.1. Extraction

Ten cloves from ten bulbs per accession were peeled, immediately immersed in liquid nitrogen, pooled within each accession, and subsequently lyophilized by freeze-drying for 48 h (Labogene ScanVac CoolSafe, Allerød, Denmark) with the condenser at −95 °C and a pressure of 0.001 mbar. Lyophilized samples were ground to powder (0.2 mm) using an ultra-centrifugal mill (Retsch ZM200, Haan, Germany) and stored in 50 mL conical tubes. Samples for total phenolic content and antioxidant capacity were weighed (75 mg) into a 2 mL centrifuge tube and extracted with 1.5 mL methanol (HPLC grade, J.T.Baker, Deventer, The Netherlands) and water (HPLC grade, VWR, Leuven, Belgium) (50:50, *v*/*v*) at 30 °C for 30 min in an ultrasonic bath (MRC 250H, Holon, Israel). Four independent technical replicates were analyzed for each accession. The samples were centrifuged at 16,000× *g* for 10 min (Domel Centric 350, Železniki, Slovenia), and the supernatant was filtered through a 0.22 µm nylon filter.

Samples for the analysis of allicin content were prepared by weighing 75 mg of dried sample and extracting it with 1.5 mL of cold methanol (HPLC grade, J.T.Baker, Deventer, The Netherlands) and water (HPLC grade, VWR, Leuven, Belgium) (70:30, *v*/*v*) at room temperature (25 °C). After adding the extraction solvent, the samples were stirred using a vortex (Biosan, V-1 plus, Riga, Latvia) for 1 min. The extracts were centrifuged at 16,000× *g* for 10 min and filtered through a 0.22 µm nylon filter before HPLC-DAD analysis. For statistical analyses, values obtained from the four technical replicates were averaged, and the accession mean was used as the experimental unit.

#### 4.3.2. Measurement of Total Phenolic Content

Total phenolic content (TPC) was determined by the Folin–Ciocalteu assay according to Singleton and Rossi (1965) [46]. Briefly, 20 µL of the methanolic extract was mixed with 140 µL of freshly prepared 0.2 M Folin–Ciocalteu reagent (Reagecon, County Clare, Ireland). After 1 min, 140 µL of sodium carbonate (p.a., Gram-Mol, Zagreb, Croatia) (6%) was added. The absorbance was read at 750 nm after 60 min of reaction time at 25 °C (Tecan Infinite 200 Pro M Nano+, Männedorf, Switzerland). The results were calculated against a standard curve of gallic acid (y = 19.45x + 0.0473; serial dilutions of gallic acid—20, 40, 60, 80, 100 mg/L (Sigma-Aldrich, St. Louis, MO, USA); coefficient of determination R^2^ = 0.9992). A reagent blank (extraction solvent processed identically to the samples) was included with every batch, and its absorbance was subtracted prior to quantification. The linearity and recovery of this assay were previously validated by our group (12.5–250 mg/L gallic acid; R^2^ = 0.9999; recovery 102.0 ± 2.9%) [47]. The results were expressed as mg of gallic acid equivalents per 100 g of dry weight (mg GAE/100 g DW). Methanol was used as a blank.

#### 4.3.3. Determination of Antioxidant Capacity

The antioxidant capacity was evaluated using the ferric-reducing antioxidant power (FRAP) assay and the DPPH radical scavenging activity assay. The FRAP assay was performed according to Benzie and Strain (1996), with some modifications [48]. Briefly, 100 µL of the methanolic extract was mixed with 200 µL of freshly prepared FRAP reagent made by mixing acetate buffer, TPTZ (98%, Alfa Aesar, Karlsruhe, Germany) and FeCl_3_·6H_2_O (pro analysi, Kemika, Zagreb, Croatia) solutions (10:1:1, *v*/*v*), and after 10 min of reaction time at 25 °C, the antioxidant capacity was measured by reading the absorbance at 593 nm (Tecan Infinite 200 Pro M Nano+, Männedorf, Switzerland). The FRAP values were calculated against a Trolox (Sigma-Aldrich, St. Louis, MO, USA) calibration curve (y = 7.8989x − 0.0179; serial dilutions of Trolox—20, 40, 60, 80, 100 µM; coefficient of determination, R^2^ = 0.9999). A reagent blank was included with every batch and subtracted from sample absorbance before quantification. The linearity and recovery of the FRAP assay under this protocol were previously validated at 101.8 ± 1.6% (20–250 µM Fe^2+^; R^2^ = 0.9999) [47]. All values are expressed as µmol of Trolox equivalents per 100 g of dry weight (µmol TE/100 g DW). The DPPH radical scavenging activity assay was performed according to Brand-Williams et al. (1995), with slight modifications [49]. Briefly, 100 µL of the methanolic extract sample was mixed with 200 µL of freshly prepared 0.1 mM DPPH radical (Sigma-Aldrich, St. Louis, MO, USA) made by dissolving 10 mg of DPPH in 250 mL of 100% methanol. After 30 min of reaction time at 25 °C, DPPH radical scavenging ability was evaluated by reading the absorbance at 517 nm (Tecan Infinite 200 Pro M Nano+, Männedorf, Switzerland). DPPH radical scavenging ability values were calculated against a standard curve of Trolox (serial dilutions of Trolox—20, 40, 60, 80, 100 µM; coefficient of determination, R^2^ = 0.9998). A reagent blank was included with every batch and subtracted from sample absorbance before quantification. The linearity and recovery of the DPPH assay under this protocol were previously validated at 103.7 ± 1.2% (2–100 µM Trolox; R^2^ = 0.9995) [47]. Values are expressed as µmol of Trolox equivalents per 100 g of dry weight (µmol TE/100 g DW). Methanol was used as a blank.

#### 4.3.4. Quantification of Allicin

Reversed-phase HPLC analysis of allicin content was performed by injecting 10 µL of the sample on a 4.6 × 50 mm C18, 2.7 µm core–shell column (Agilent Poroshell EC-C18, Palo Alto, CA, USA), held at 35 °C, with a flow rate of the mobile phase (50:50 methanol: water, *v*/*v*) set at 1 mL/min. The HPLC system consisted of a binary solvent delivery unit (two Shimadzu Nexera LC-40DX3 pumps, Kyoto, Japan), autosampler (Shimadzu SIL-40CX3, Kyoto, Japan), column oven (Shimadzu CTO-40C, Kyoto, Japan), and DAD detector (Shimadzu SPD-M40, Kyoto, Japan). Allicin was identified and quantified by comparing its retention time and peak area at 240 nm to a serial dilution of an allicin certified reference standard (Sigma-Aldrich, St. Louis, MO, USA). Calibration was performed using an external standard curve prepared from serial dilutions of the allicin reference standard at 10, 25, 50, 75, and 100 mg/L, each injected in quadruplicate. The resulting calibration curve (y = 109.14x − 147.57; R^2^ = 0.9997) was linear over this range, with instrument precision (%RSD of peak area, *n* = 4) between 0.48% and 4.47% across the five levels. The limit of detection (LOD) and limit of quantification (LOQ), calculated from the residual standard deviation of the calibration line and its slope (LOD = 3.3σ/S; LOQ = 10σ/S), were 1.84 and 5.58 mg/L, respectively. A solvent blank was injected between sample batches to confirm the absence of interference at the allicin retention time. Accuracy was assessed by a matrix spike-recovery experiment (10 mg/L spike level, *n* = 4), giving a mean recovery of 102.86 ± 1.11%. A representative chromatogram is shown in the Supplementary Materials.

### 4.4. Data Analysis

The Shannon information index [50] was used to quantify phenotypic diversity of qualitative traits according to:

$$H_{j}=-\sum_{i=1}^{k}p_{i}\log_{2}\left(p_{i}\right),$$

where *p_i_* is the frequency of the *i*th phenotypic class and *k* is the total number of phenotypic classes.

The Shannon index was used to estimate total diversity (H_T_), diversity among accessions within each accession provenance group (H_R_), average diversity among accessions within accession provenance groups (H_RA_), and average diversity within accessions (H_L_). The proportion of diversity among accession provenance groups was calculated as 100 × (1 − H_RA_/H_T_), whereas the proportion of diversity within accession provenance groups was calculated as 100 × (H_RA_/H_T_). Similarly, the proportions of diversity among and within accessions across all accession provenance groups were calculated as 100 × (1 − H_L_/H_T_) and 100 × (H_L_/H_T_), respectively. Within each region, the proportions of diversity among and within accessions were calculated as 100 × (1 − H_L_/H_R_) and 100 × (H_L_/H_R_), respectively.

Uncertainty associated with diversity estimates was assessed by nonparametric bootstrap resampling of accessions within regions (1000 replicates). Bootstrap-derived 95% confidence intervals were calculated for all Shannon diversity indices and diversity-partitioning estimates and are presented in the corresponding tables. All analyses were performed in R (Version 1.2.1) [51], using functions from the dplyr package [52] for data manipulation and bootstrap processing.

Principal component analysis (PCA) was performed using the PRINCOMP procedure in SAS 9.4M9 (SAS Institute, Cary, NC, USA) through SAS OnDemand for Academics to summarise multivariate variation in seven qualitative morphological traits. Because individual plants within the same accession could exhibit different states for a given trait, the frequency of each trait state was calculated for each accession based on the 10 sampled plants. These accession-level frequencies were used as quantitative variables in the PCA rather than the original categorical observations. Thus, the PCA was used to summarise patterns of variation in the relative frequencies of morphological trait states among garlic accessions. K-means cluster analysis was performed using the high-performance clustering (HPCLUS) procedure in SAS (SAS Institute, Cary, NC, USA). The optimal number of clusters was determined using the aligned box criterion (ABC), which evaluates candidate numbers of clusters based on the gap statistic.

Spearman rank correlation coefficients were calculated to assess associations among the biochemical properties TPC, FRAP, DPPH, and allicin using the CORR procedure in SAS (SAS Institute, Cary, NC, USA). Correlation analyses were performed separately for each vegetation year. For each year, 95% confidence intervals for Spearman correlation coefficients were obtained using 1000 bootstrap resamplings. To account for multiple pairwise comparisons, p-values were adjusted using the Holm method [53]. For each correlation, the Spearman correlation coefficient, 95% bootstrap confidence interval, and Holm-adjusted p-value were reported.

The obtained data were analysed using linear mixed models implemented in the MIXED procedure of SAS (SAS Institute, Cary, NC, USA). Morphological group (*n* = 2), accession provenance (*n* = 4), vegetation year (*n* = 2), and their two- and three-way interactions were included as fixed effects, while accession nested within morphological group × accession provenance was included as a random effect. The model was specified as:

Y_ijkl_ = μ + G_i_ + R_j_ + V_k_ + (GR)_ij_ + (GV)_ik_ + (RV)_jk_ + (GRV)_ijk_ + A_l(ij)_ + ε_ijkl_,

where Y_ijkl_ is the observed response, μ is the overall mean, G_i_, R_j_, and V_k_ are the fixed effects of morphological group, accession provenance, and vegetation year, respectively; (GR)_ij_, (GV)_ik_, (RV)_jk_, and (GRV)_ijk_ represent the corresponding two- and three-way interactions; A_l(ij)_ is the random effect of accession nested within morphological group × accession provenance; and ε_ijkl_ is the residual error. Denominator degrees of freedom were estimated using the Kenward–Roger method (DDFM = KR). Each accession was represented by a single field plot in each vegetation year. Consequently, potential accession effects cannot be completely separated from small-scale spatial variation within the field, and the absence of replicated field plots represents a limitation of the experimental design. When significant interactions were detected, the corresponding main effects were not interpreted independently. Instead, simple effects were examined using the SLICE option in PROC MIXED, allowing the effect of one factor to be tested separately at each level of the interacting factor. For significant simple effects, pairwise differences among estimated marginal means were assessed using Tukey-adjusted comparisons at *p* ≤ 0.05. Results are presented as estimated marginal means ± standard errors (SE), with different lowercase letters indicating significant differences among means according to Tukey’s HSD test.

## 5. Conclusions

Although four qualitative traits clearly distinguished the two garlic morphotypes, they showed no significant differences in TPC, FRAP, DPPH, or allicin content. Accession provenance was associated with differences in TPC and FRAP: accessions from Istria and Kvarner reached the highest values (in 2018/2019, 820.8 mg GAE/100 g DW and 354.6 µmol TE/100 g DW), significantly exceeding Continental Croatia (494.1 and 180.4, respectively), although this pattern varied between the two vegetation years. A year-to-year difference was observed in DPPH antioxidant capacity and allicin content. TPC was positively correlated with both FRAP and DPPH antioxidant capacities in both vegetation years.

Taken together, these results show that morphotype is not a reliable predictor of biochemical quality in garlic: the two morphological groups did not differ in TPC, FRAP, DPPH, or allicin content, whereas accession provenance and growing season did. From a practical standpoint, this means that morphological and biochemical characterization are complementary rather than interchangeable tools and that selection for a desired biochemical profile cannot rely on bulb or bolting phenotype alone. The higher TPC of accessions from Istria and Kvarner, together with the high private genotypic richness previously reported for this region using molecular markers [33], identifies Istria and Kvarner as a particularly valuable source of material for conservation and for breeding aimed at antioxidant quality. The differences observed between the two vegetation years suggest that the growing season may influence DPPH and allicin content; however, as these observations are based on only two seasons, they should be interpreted as an indication of a possible seasonal trend rather than as a definitive effect, and multi-year evaluation would be needed to characterize it in more detail. Screening a collection of this size for both morphological and biochemical traits nonetheless provides a practical basis for prioritizing accessions in germplasm management and breeding programs.

## Figures and Tables

**Figure 1:**
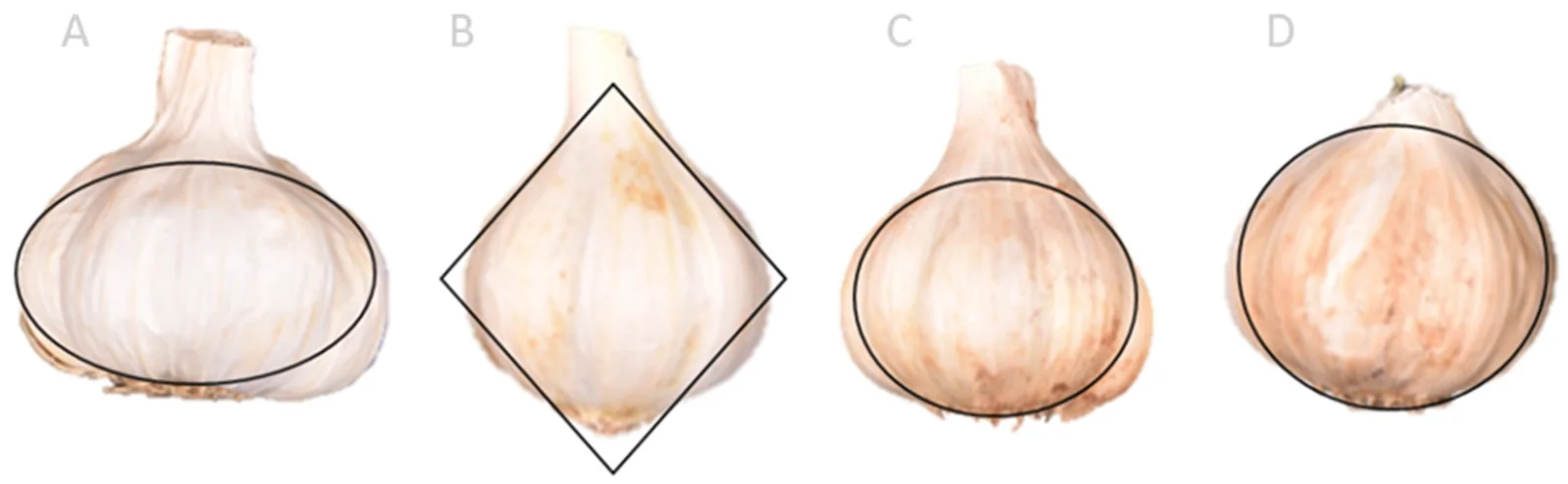
Shape of mature dry bulbs: flat globe Ql1 2 (**A**), rhomboid Ql1 3 (**B**), broad oval Ql1 4 (**C**), and globe Ql1 5 (**D**).

**Figure 2:**
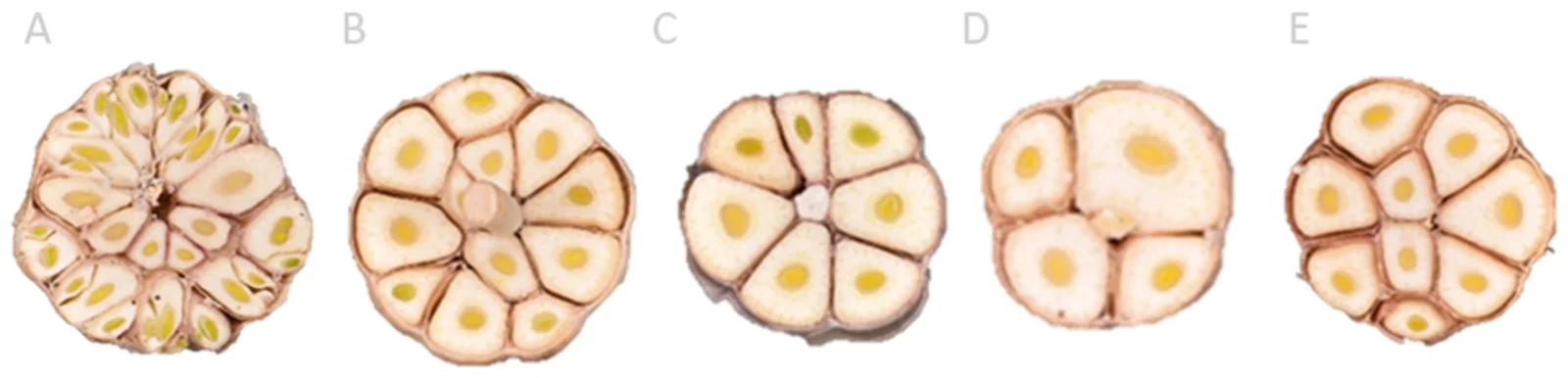
Bulb structure type: regular multi-fan groups (or multi-shelled) Ql5_1 (**A**), regular two-fan groups Ql5_2 (**B**), regular multi-cloved radial Ql5_3 (**C**), regular quadruple Ql5_4 (**D**), irregular Ql5_6 (**E**).

**Figure 3:**
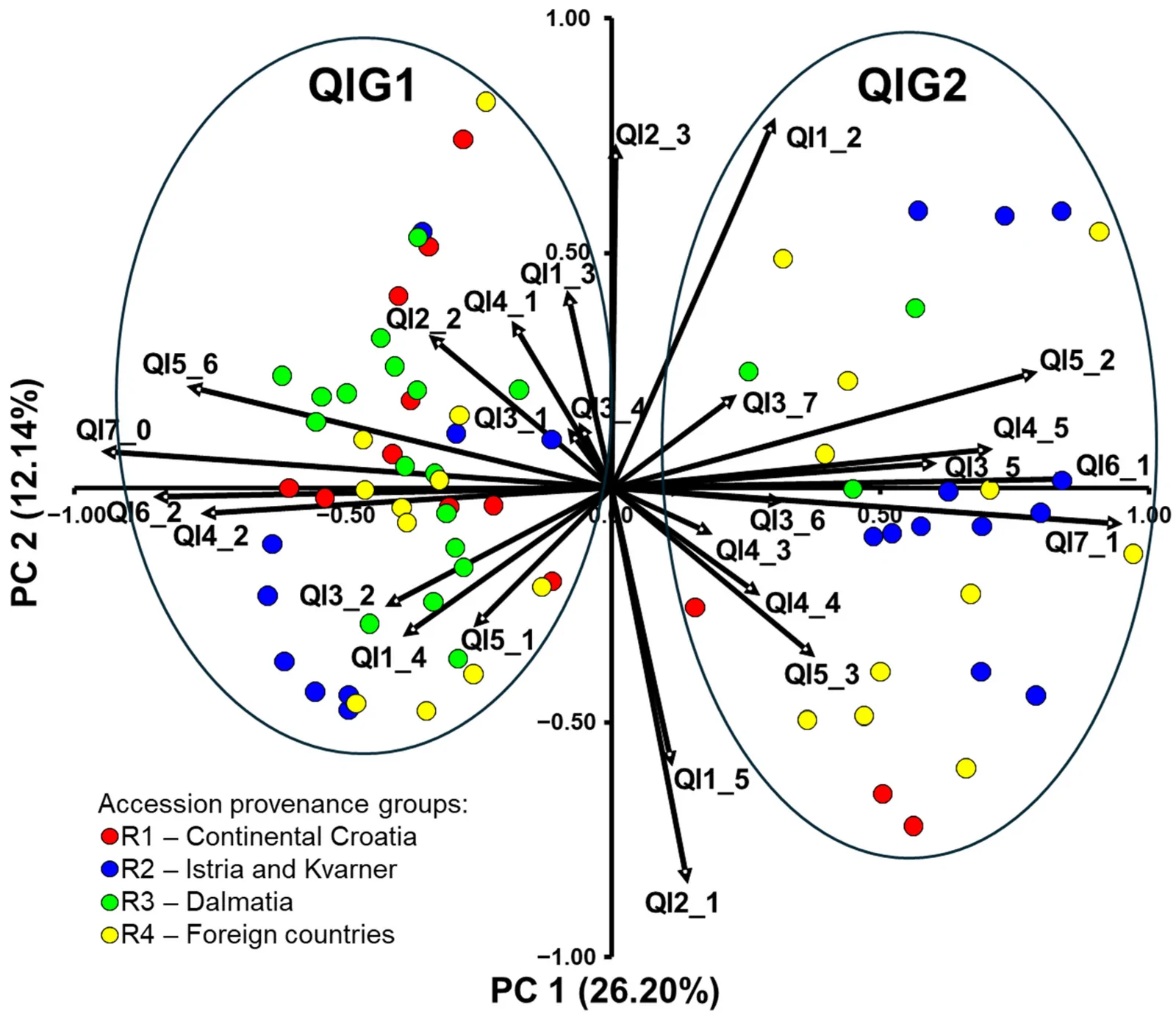
PCA plot of 2 garlic morphotype groups and their distinctive traits. Abbreviations: Ql-qualitative trait; Variables: shape of mature dry bulbs—flat globe Ql1_2, shape of mature dry bulbs—rhomboid Ql1_3, shape of mature dry bulbs—broad oval Ql1_4, shape of mature dry bulbs—globe Ql1_5, shape of mature garlic bulb—circular, basal plate prominent Ql2_1, shape of mature garlic bulb—heart-shaped, basal plate retracted Ql2_2, shape of mature garlic bulb—broadly ovate, basal plate even Ql2_3, outer skin color of compound bulb—white Ql3_1, outer skin color of compound bulb—cream Ql3_2, outer skin color of compound bulb—white stripes Ql3_4, outer skin color of compound bulb—light violet Ql3_5, outer skin color of compound bulb—violet Ql3_6, outer skin color of compound bulb—dark violet Ql3_7, skin color of the clove—white Ql4_1, skin color of the clove—yellow and light brown Ql4_2, skin color of the clove—brown Ql4_3, skin color of the clove—red Ql4_4, skin color of the clove—violet Ql4_5, bulb structure type—regular multi-fan groups (or multi-shelled) Ql5_1, bulb structure type—regular two-fan groups Ql5_2, bulb structure type—regular multi-cloved radial Ql5_3, bulb structure type—regular quadruple Ql5_4, bulb structure type—irregular Ql5_6, shape of the compound bulb in horizontal section—circular Ql6_1, shape of the compound bulb in horizontal section—elliptic Ql6_2, and ability to flower—no Ql7_0, ability to flower—yes Ql7_1.

**Figure 4:**
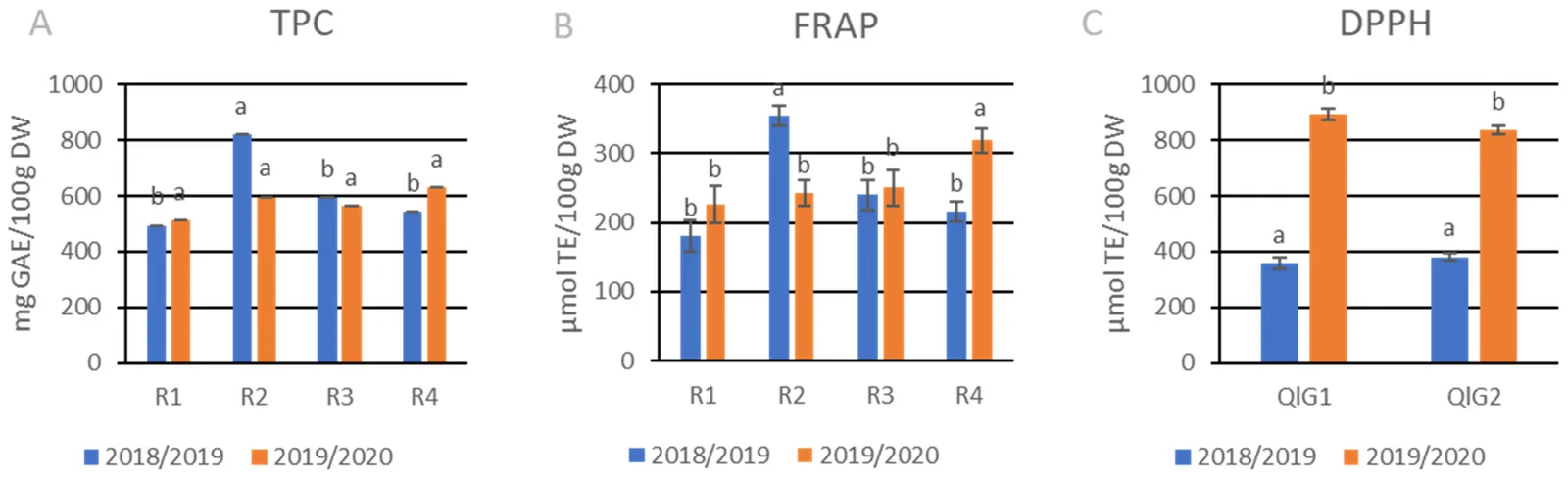
Significant interaction effects on garlic biochemical parameters are shown as estimated marginal means (LS-means) ± standard error. Total phenolic content (**A**) and ferric-reducing antioxidant power (**B**) are shown by accession provenance × vegetation year, and DPPH radical-scavenging activity (**C**) by morphological group × vegetation year, for the two vegetation years (2018/2019 and 2019/2020). Simple effects of the significant interactions were analyzed using the SLICE option in the MIXED procedure (SAS), and pairwise comparisons were performed using Tukey’s HSD test at *p* ≤ 0.05; for each parameter, means sharing the same letter do not differ significantly. R1, Continental Croatia; R2, Istria and Kvarner; R3, Dalmatia; R4, non-Croatian accessions. QlG1 and QlG2 denote the two morphological groups.

**Table 1:** Shannon diversity indices and diversity partitioning for seven qualitative traits of 77 garlic accessions. Total diversity (H_T_), average diversity within accessions (H_LT_), average diversity among accessions within accession provenance groups (H_RA_), and the proportions of diversity partitioned among and within accessions and accession provenance groups are presented. Values in parentheses indicate 95% bootstrap confidence intervals.

Trait ^a^	H_T_	H_LT_	Diversity (%) ^b^		H_RA_	Diversity (%) ^c^	
			Among Accessions	Within Accessions		Among Accession Provenance Groups	Within Accession Provenance Groups
Q11	1.623 (1.54–1.68)	0.903 (0.82–0.98)	44.38 (38.79–49.66)	55.62 (50.34–61.21)	1.596 (1.48–1.63)	1.68 (1.06–6.54)	98.32 (93.46–98.94)
Q12	1.284 (1.19–1.36)	0.679 (0.58–0.79)	47.12 (39.56–53.86)	52.88 (46.14–60.44)	1.238 (1.12–1.30)	3.63 (1.82–9.66)	96.37 (90.34–98.18)
Q13	1.889 (1.73–2.01)	0.709 (0.59–0.82)	62.45 (56.51–67.49)	37.55 (32.51–43.49)	1.696 (1.52–1.74)	10.22 (8.07–17.57)	89.78 (82.43–91.93)
Q14	1.924 (1.73–2.05)	0.770 (0.65–0.90)	59.95 (53.52–65.32)	40.05 (34.68–46.48)	1.763 (1.52–1.84)	8.38 (6.46–16.66)	91.62 (83.34–93.54)
Q15	1.867 (1.71–1.96)	0.516 (0.42–0.61)	72.35 (67.25–77.13)	27.65 (22.87–32.75)	1.722 (1.46–1.79)	7.75 (5.71–18.76)	92.25 (81.24–94.29)
Q16	0.999 (0.98–1.00)	0.633 (0.55–0.72)	36.62 (28.00–45.10)	63.38 (54.90–72.00)	0.965 (0.90–0.98)	3.37 (1.32–8.66)	96.63 (91.34–98.68)
Q17	0.963 (0.87–1.00)	0.133 (0.08–0.19)	86.23 (80.02–91.42)	13.77 (8.58–19.98)	0.853 (0.67–0.93)	11.44 (4.27–28.86)	88.56 (71.14–95.73)

^a^ Traits: Ql1—shape of mature dry bulbs; Ql2—shape of mature garlic bulb; Ql3—outer skin color of compound bulb; Ql4—skin color of the clove; Ql5—bulb structure type; Ql6—shape of the compound bulb in horizontal section; Ql7—ability to flower. ^b^ Diversity among accessions (%) = 100 × (1 − H_LT_/H_T_); diversity within accessions (%) = 100 × (H_LT_/H_T_). ^c^ Diversity among accession provenance groups (%) = 100 × (1 − H_RA_/H_T_); diversity within accession provenance groups (%) = 100 × (H_RA_/H_T_).

**Table 2:** Significance of the effects of morphological group, accession provenance, vegetation year, and their interactions on garlic biochemical parameters. TPC: total phenolic content, FRAP: ferric reducing antioxidant power; DPPH: 2,2-diphenyl-1-picrylhydrazyl.

Source of Variation	TPC	FRAP	DPPH	Allicin
	mg GAE/100 g DW	µmol TE/100 g DW	µmol TE/100 g DW	mg/100 g DW
Morphological group	ns	ns	ns	ns
Accession provenance	***	**	ns	ns
Year	ns	ns	***	***
Group × Accession provenance	ns	ns	ns	ns
Group × Year	ns	ns	*	ns
Accession provenance × Year	***	***	ns	ns
Group × Accession provenance × Year	ns	ns	ns	ns

Significance level (*p*): * *p* < 0.05,** *p* < 0.01, *** *p* < 0.001, ns = not significant.

**Table 3:** Spearman rank correlations among biochemical properties of garlic accessions in two vegetation years. Correlations were calculated using the same 77 accessions in each vegetation year. Values are Spearman’s rank correlation coefficients (ρ), with 95% bootstrap confidence intervals (CI) and Holm-adjusted *p*-values for multiple comparisons.

Year	Correlation	Spearman’s ρ	95% CI	Holm-Adjusted *p*
2018/2019	TPC/FRAP	0.892	0.88–0.96	<0.000
2018/2019	TPC/DPPH	0.456	0.27–0.63	<0.000
2018/2019	TPC/allicin	−0.085	−0.33–0.14	0.929
2018/2019	FRAP/DPPH	0.492	0.29–0.61	<0.000
2018/2019	FRAP/allicin	0.040	−0.22–0.22	0.727
2018/2019	DPPH/allicin	−0.233	−0.35–0.05	0.123
2019/2020	TPC/FRAP	0.669	0.57–0.79	<0.000
2019/2020	TPC/DPPH	0.360	0.14–0.52	0.003
2019/2020	TPC/allicin	0.373	0.12–0.49	0.002
2019/2020	FRAP/DPPH	0.550	0.32–0.71	<0.000
2019/2020	FRAP/allicin	0.382	0.25–0.61	0.002
2019/2020	DPPH/allicin	0.182	−0.04–0.38	0.114

## Data Availability

The datasets and SAS analysis code generated and analyzed in this study are available in Zenodo, https://doi.org/10.5281/zenodo.22690365.

## Supplementary Materials

The following supporting information can be downloaded at https://www.mdpi.com/article/10.3390/plants15192906/s1: Figure S1. A representative chromatogram showing the allicin peak; Figure S2. Average monthly precipitation (A), air temperature (B), and relative humidity (C) at the IPTPO meteorological station during the two experimental growing seasons (2018/2019 and 2019/2020); Table S1. List of accessions included in the research; Table S2. Average diversity among accessions within regions (H_R_), average diversity within accessions (H_L_), and proportions of diversity among and within accessions for seven qualitative traits in four geographic regions. Values in parentheses indicate 95% bootstrap confidence intervals.
