# Supplementary material for: Biochemical Quality of Garlic Differs by Accession Provenance and Growing Season Rather than Morphotype
Source: Plants (Basel). 2026 Sep 23;15(19):2906. doi: 10.3390/plants15192906 (PMC13667241; doi:10.3390/plants15192906)
Supplement: Supplementary file 1 [file plants-15-02906-s001.zip › Table S2.pdf]

Table S2. Average diversity among accessions within regions (H_R), average diversity within accessions (H_L), and proportions of diversity among and within accessions for seven qualitative traits in four geographic regions. Values in parentheses indicate 95% bootstrap confidence intervals.

Trait[a]	Region[b]	H_R	H_L	Diversity (%)[c]	
				Among accessions	Within accessions
**Ql1**	R1	1.744 (1.44-1.86)	1.072 (0.85-1.27)	38.54 (22.45-50.41)	61.46 (49.59-77.55)
	R2	1.614 (1.48-1.66)	0.858 (0.70-1.02)	46.83 (34.19-55.67)	53.17 (44.33-65.81)
	R3	1.565 (1.46-1.58)	0.989 (0.89-1.09)	36.82 (29.11-42.20)	63.18 (57.80-70.89)
	R4	1.461 (1.19-1.60)	0.770 (0.60-0.94)	47.31 (32.42-58.57)	52.69 (41.43-67.58)
**Ql2**	R1	1.244 (1.00-1.36)	0.743 (0.44-1.02)	40.27 (18.24-60.49)	59.73 (39.51-81.76)
	R2	1.153 (1.01-1.24)	0.635 (0.45-0.83)	44.92 (30.35-59.02)	55.08 (40.98-69.65)
	R3	1.244 (0.95-1.43)	0.846 (0.65-1.03)	32.03 (18.16-43.36)	67.97 (56.64-81.84)
	R4	1.310 (1.06-1.46)	0.534 (0.34-0.73)	59.21 (42.09-72.30)	40.79 (27.70-57.91)
**Ql3**	R1	1.351 (1.04-1.51)	0.761 (0.55-0.95)	43.68 (23.80-56.64)	56.32 (43.36-76.20)
	R2	1.969 (1.58-2.14)	0.705 (0.44-0.99)	64.19 (49.75-75.32)	35.81 (24.68-50.25)
	R3	1.665 (1.40-1.76)	0.692 (0.49-0.87)	58.45 (46.28-69.16)	41.55 (30.84-53.72)
	R4	1.799 (1.52-1.93)	0.699 (0.49-0.91)	61.16 (48.06-71.03)	38.84 (28.97-51.94)
**Ql4**	R1	1.594 (1.18-1.83)	0.902 (0.61-1.15)	43.43 (28.80-54.16)	56.57 (45.84-71.20)
	R2	2.077 (1.79-2.19)	0.939 (0.68-1.22)	54.78 (42.75-63.75)	45.22 (36.25-57.25)
	R3	1.486 (0.95-1.80)	0.585 (0.37-0.82)	60.66 (37.66-73.61)	39.34 (26.39-62.34)
	R4	1.893 (1.49-2.07)	0.693 (0.47-0.90)	63.38 (50.83-73.02)	36.62 (26.98-49.17)
**Ql5**	R1	1.700 (1.06-1.91)	0.481 (0.25-0.71)	71.73 (52.15-83.93)	28.27 (16.07-47.85)
	R2	1.623 (1.35-1.72)	0.585 (0.40-0.78)	63.96 (53.11-73.54)	36.04 (26.46-46.89)
	R3	1.619 (0.95-1.92)	0.380 (0.21-0.57)	76.50 (56.89-85.94)	23.50 (14.06-43.11)
	R4	1.947 (1.65-2.04)	0.592 (0.39-0.79)	69.60 (59.52-77.59)	30.40 (22.41-40.48)
**Ql6**	R1	0.971 (0.81-1.00)	0.611 (0.38-0.80)	37.11 (10.29-59.93)	62.89 (40.07-89.71)
	R2	0.996 (0.90-1.00)	0.471 (0.29-0.64)	52.70 (35.02-70.08)	47.30 (29.92-64.98)
	R3	0.944 (0.86-0.99)	0.810 (0.67-0.93)	14.18 (4.74-24.64)	85.82 (75.36-95.26)
	R4	0.950 (0.83-1.00)	0.648 (0.48-0.81)	31.84 (16.79-46.81)	68.16 (53.19-83.21)
**Ql7**	R1	0.741 (0.20-0.98)	0.128 (0.00-0.27)	82.77 (42.36-100.00)	17.23 (0.00-57.64)
	R2	0.990 (0.83-1.00)	0.162 (0.05-0.28)	83.59 (69.48-94.34)	16.41 (5.66-30.52)
	R3	0.680 (0.26-0.92)	0.155 (0.05-0.29)	77.18 (38.74-92.31)	22.82 (7.69-61.26)
	R4	1.000 (0.82-1.00)	0.085 (0.02-0.17)	91.47 (82.71-97.86)	8.53 (2.14-17.29)

[a]Traits: Traits: Ql1 – shape of mature dry bulbs; Ql2 – shape of mature garlic bulb; Ql3 – outer skin color of compound bulb; Ql4 – skin color of the clove; Ql5 – bulb structure type; Ql6 – shape of the compound bulb in horizontal section; Ql7 – ability to flower.

[b]Regions: R1 – Continental Croatia; R2 – Istria; R3 – Dalmatia; R4 – Foreign countries.

[c]Diversity among accessions (%) = 100 × (1 − H_L/H_R); diversity within accessions (%) = 100 × (H_L/H_R).
